# Central Control of Circadian Phase in Arousal-Promoting Neurons

**DOI:** 10.1371/journal.pone.0067173

**Published:** 2013-06-24

**Authors:** Carrie E. Mahoney, Judy McKinley Brewer, Eric L. Bittman

**Affiliations:** 1 Neuroscience and Behavior Program, University of Massachusetts, Amherst, Massachusetts, United States of America; 2 Department of Biology, University of Massachusetts, Amherst, Massachusetts, United States of America; University of Texas Southwestern Medical Center, United States of America

## Abstract

Cells of the dorsomedial/lateral hypothalamus (DMH/LH) that produce hypocretin (HCRT) promote arousal in part by activation of cells of the locus coeruleus (LC) which express tyrosine hydroxylase (TH). The suprachiasmatic nucleus (SCN) drives endogenous daily rhythms, including those of sleep and wakefulness. These circadian oscillations are generated by a transcriptional-translational feedback loop in which the *Period* (*Per*) genes constitute critical components. This cell-autonomous molecular clock operates not only within the SCN but also in neurons of other brain regions. However, the phenotype of such neurons and the nature of the phase controlling signal from the pacemaker are largely unknown. We used dual fluorescent *in situ* hybridization to assess clock function in vasopressin, HCRT and TH cells of the SCN, DMH/LH and LC, respectively, of male Syrian hamsters. In the first experiment, we found that *Per1* expression in HCRT and TH oscillated in animals held in constant darkness with a peak phase that lagged that in AVP cells of the SCN by several hours. In the second experiment, hamsters induced to split their locomotor rhythms by exposure to constant light had asymmetric *Per1* expression within cells of the middle SCN at 6 h before activity onset (AO) and in HCRT cells 9 h before and at AO. We did not observe evidence of lateralization of *Per1* expression in the LC. We conclude that the SCN communicates circadian phase to HCRT cells via lateralized neural projections, and suggests that *Per1* expression in the LC may be regulated by signals of a global or bilateral nature.

## Introduction

Temporal coordination of mammalian physiology and behavior is maintained by the suprachiasmatic nucleus of the hypothalamus (SCN; [1]). Neurons of the SCN contain a molecular clock whose intrinsic transcriptional –translational loop operates with a ∼24-hour period. Circadian phase is marked by the expression of canonical clock genes whose transcription is sensitive to environmental cues, among which light is the principal *zeitgeber*. The endogenous pacemaker is thus entrained by the periodic environment and in turn regulates the phase of subordinate oscillators elsewhere in the brain and in the peripheral organs 2–13.

Among functions regulated by the SCN, the sleep-wake cycle is particularly prominent and biologically significant [14]. Hypocretins produced exclusively in the perifornical dorsomedial hypothalamus act to promote wakefulness not only through ascending projections, but also by exciting arousal-promoting midbrain regions, including the locus coeruleus [15]–[20]. Hypocretin signaling 21,22 as well as an intact SCN [14] are necessary for normal consolidation and temporal patterning of sleep. Activation of *HCRT*-expressing cells is modulated by the SCN [23]–[25]. Although the phenotype of some extra-SCN neuronal oscillators has been established [26]–[32], the expression of clock genes in neurons that regulate sleep and wakefulness is not established and their dependence upon the SCN pacemaker is unclear. Our first experiment was designed to determine whether clock genes are expressed in a circadian pattern within two arousal-promoting cell types, the hypocretin cells of the lateral/dorsomedial hypothalamus (LH/DMH) and the tyrosine hydroxylase (TH)-expressing cells of the locus coeruleus (LC).

Our results from free running hamsters established rhythmicity of clock gene expression in HCRT and TH expressing cells of the LH/DMH and LC, respectively. These findings raised the question of whether rhythmicity in these neurons was regulated by the SCN, and if so whether lateralized or diffusible signals might contribute. In order to address this question, we took advantage of the finding that upon prolonged exposure to constant light, most Syrian hamsters adopt a bimodal activity pattern [33]. Remarkably, clock gene transcription and neuronal activation of the left and right SCN enter an antiphase relationship in these circumstances [34]. This split preparation has proven useful in establishing the role of lateralized pacemaker projections in physiologically relevant circuits as opposed to diffusible humoral signals [35], [36]. We used behaviorally split Syrian hamsters to determine whether lateralized neuronal timing signals from the SCN set the phase of clock gene expression within HCRT neurons of the LH/DMH and TH-expressing neurons of the LC. If descending projections of the SCN control the function of wake-promoting regions, we expect to find asymmetry of *Per1* expression in hypocretin or TH cells in the LH/DMH or LC respectively.

## Methods

### Animals and their Treatment

For experiment 1, adult male Syrian hamsters (Mesocricetus auratus, LVG strain) born and raised in 14 L: 10D were allowed ad libitum access to food and water throughout the experiment. All procedures were approved by the University of Massachusetts Institutional Animal Care and Use Committee (Assurance Number: A3551-01). As young adults, hamsters were transferred to constant darkness for 10 days. Activity in a running wheel (16.5 cm diameter) was monitored by computer (ClockLab software, Actimetrics, Evanston, IL). A least-squares regression line was fit to the activity onsets in order to estimate period and establish the time of onset of subjective night (CT12). Hamsters were killed at CT 3, 9, 12.5 or 22 (n = 5 per phase). Figure S1A depicts a representative actogram of an animal killed at CT12.5. The brains of these animals were removed immediately after decapitation under dim red light (<1 lux), and were rapidly frozen and stored at −80°C.

For experiment 2, Syrian hamsters were transferred as young adults (approximately 10 weeks of age) to a cage with access to a running wheel and maintained in LL (white fluorescent light; ∼200 lux at cage level) for 15±4 weeks. In addition, Chi square periodograms were analyzed to verify split locomotor patterns. Hamsters were classified as split if two activity bouts occurred each 24-hour cycle at intervals of approximately 12 h. A stable split was achieved approximately 8 weeks from transition into LL (range of 4 to 13 weeks; see figure S1B and [37]). The morning and evening activity bouts were designated as described by Pittendrigh and Daan [33]. Briefly, it is possible to identify which of the bouts can be extrapolated to the phase of activity onset (AO) before the split occurs, and this evening activity bout was used to predict the kill times. Twenty-six animals were killed 7 weeks after a stable split was established. Groups of 5 hamsters were rapidly decapitated at 0, 3, 6 (n = 5 each), or 9 (n = 6) hours before AO. Another five hamsters that displayed a single activity bout per 24 hours (non-split controls) were killed at 3 h before AO. The brains of these animals were collected and stored as described above. Two sets of DMH sections were not used in quantification due to loss during assay (n = 5 for 9 h, 6 h, 0 h before AO and n = 4 for 3 h before AO).

### *In situ* Hybridization

Brains collected in both experiments were equilibrated to −20°C, notched on one side and sectioned in the coronal plane on a cryostat at a thickness of 20 µm before processing for in situ hybridization in order to determine levels of Per1 expression within vasopressin (AVP) cells of the SCN, HCRT cells of the LH/DMH, and tyrosine hydroxylase (TH) cells of the LC.

*Probe Preparation.* In order to prepare template for *HCRT* probes, the following primers were used to amplify a 0.29 kb RNA transcript isolated from Syrian hamster whole brain: FWD-5′-CAGCCTCTGCCCGACTGCTGTCGCCAGAAG-3′ and REV-5′-GACTCCGGAGCCTCCCCGGGGTGCTAAAGC-3′. The PCR product was gel purified (QiaxeII Agarose Gel Extraction kit), ligated into pGEM-T Easy Vector (Promega, Madison, WI) and transformed into XL 10-Gold Ultracompetent Cells (Stratagene). Transformed colonies were grown and plasmids were isolated and sequenced (Genewiz, Inc, South Plainfield, NJ) to confirm template sequence for production of riboprobes. *In situ* hybridization performed using ^35^S-labeled probe confirmed the expected anatomical distribution of *HCRT* expression in Syrian hamster brain (figure S2).

pBluescript II SK+ plasmid containing the hamster *period 1* (*haper1*) cDNA sequence (AF249882 nt 215–1336, homologous to AF022992 nt 337–1120, ∼730 bp), was linearized with *HindIII*, for antisense, or *SmaI*, for sense probes. The antisense probe was transcribed with T3 polymerase and sense probe with T7 polymerase in presence of digoxigenin-11-UTP (Roche Diagnostics). Probes were isolated using ProbeQuant-50 spin column (GEHealthCare Bio-Sciences Corp) and checked on a 1% agarose gel prior to hybridization.

In order to permit colocalization with *Per1*, transcription of the remaining probes was carried out in the presence of fluorescein-12-UTP (Roche Diagnostics). For *AVP*, pGEM3 plasmid containing exon C of rat vasopressin cDNA (*rAVP*; [38] originally from Sherman Thomas G. University of Pittsburgh) was linearized with *EcoR*1 or *Hind*III for antisense and sense probe transcription, respectively. SP6 and T7 polymerase were used to transcribe antisense and sense probes (∼293 nt), respectively. For hypocretin, pGEM-T Easy plasmid containing the hamster hypocretin (*HCRT*) sequence (homologous to *Mus musculus* NM_010410.2, nt 187-480) was linearized with *Apa*1 for antisense or Spe-1 for sense probe preparation. SP6 and T7 polymerase were used to transcribe antisense and sense probes, respectively. For tyrosine hydroxylase, rat tyrosine hydroxylase (r*TH*; NM_012740; nt 14-1165; [39], [40] originally obtained from Donna M. Chikaraishi, Duke University School of Medicine) cDNA in pBS(-) was linearized with *Pvu1* or *HindIII* for antisense and sense probe transcription, respectively. T3 polymerase or T7 polymerase was used to transcribe antisense and sense probes, respectively.

#### Hybridization

The non-isotopic dual label *in situ* hybridization method used in this experiment was adapted from Watakabe et al. [41] and was carried out on slide mounted prehybridized brain sections. Slides were presoaked in hybridization buffer (final concentration 5xSSC, 50% Formamide, 5xDenhardt’s, 250ug/mL yeast tRNA, 500ug/mL salmon sperm DNA) for 2 hours at room temperature. Probes (400 ng/mL hybridization buffer) were heated to 80°C for 5 minutes and quenched on ice. Excess buffer was wicked off and probe was added (90uL) before the tissue was cover slipped (Hybri-slips, Sigma-Aldrich, St Louis, MO). Slides were incubated at 55°C for 36 hours.

#### Post hybridization

Coverslips were floated off in pre-warmed 5xSSC. Slides were washed in 0.2xSSC three times at 55°C, and washed in 1X Tris HCl (pH 7.5) at room temperature for 5 minutes. Tissue was incubated in 1% blocking buffer (PerkinElmer) for 1 hour at room temperature. Blocking reagent was blotted off and 230uL of anti-fluorescein-POD (1∶2000; Roche) overnight at 4°C. Slides were washed 3x in TNT (Tris-NaCl pH 7.5+0.05% Tween 20) for 10 minutes each. Tyramide Signal Amplification-plus DNP amplification (PerkinElmer) was carried out according to the suppliers’ instructions 1x 10 minutes, followed by 3 washes in TNT for 5 minutes each. Slides were incubated with 1∶2000 anti-DIG-AP (Roche) and 1∶2500 anti-DNP-Alexa488 (Roche) for 48 hours at 4°C. Slides were washed 3x in TNT for 10 minutes each at room temperature and washed once in TRIS pH 8.0 for 5 minutes at room temperature. HNPP/FR (1∶100) development was carried out 3 times at room temperature for 20 minutes each. The reaction was stopped in PBS-EDTA (2 washes at 5 minutes each). Tissue was cover slipped using CC mount (Sigma Aldrich).

### Image Acquisition

Images were captured on a Zeiss 510 microscope using FITC/Rhodamine selection (Band Pass 505–530 nm and Long Pass 560 nm). The sections were excited with an Argon and Helium-neon laser using the excitation wavelengths of 488 nm (for the Alexafluor488 fluorophore) and 543 nm (for HNPP/FR). To prevent cross-talk of the two fluorophores, images were collected using multitrack optical sections. On average, ten-1 µm thick optical slices were taken. Images were saved as layered TIFF files (.lsm). The dispersed population of the HCRT field required, on average, three images (300×300um in dimension) to capture the medial, perifornical and lateral segments (similar to [42]). Standard fluorescent beads (Molecular Probes) were imaged during each imaging session. Quantification of cell number, *Per1* intensity and colocalization was conducted using Image J (NIH software). The extent of colocalization was quantified using the Manders’ coefficient [43] within the region of interest. Colocalization within digital images is defined as the amount of overlap of two different channels within a multichannel image. To remove bias, many algorithms have been developed to quantify this overlap. The Manders’ coefficient describes the contribution of each channel to the overlap with minimal influence of fluorophore intensity [43]. A Manders’ coefficient of 0.5 or greater objectively confirms colocalization and represents 50% or more overlap of the two channels. Mean intensity within each region of interest was measured. Intensity values were normalized to the standard curve generated from the relative intensity levels of the standard fluorescent beads (Molecular Probes). In order to assess the distribution of intensities of *Per1* expression within HCRT or TH cells in experiment one, the range of intensities was calculated for all sections and for each circadian phase the percent of cells falling in the highest quartile (values greater than quartile 3), moderate (values falling within quartile 2 and 3) and low (values less than quartile 2) *Per1* intensity was determined. For experiment 2, within animal quartiles were determined to evaluate asymmetry in distribution of *Per1* intensity between sides of the brain.

### Statistical Analysis

For experiment 1, cell counts within each tissue quantified and mean *Per1* intensity levels were evaluated across phases (n = 5 per phase except 3 h before AO n = 4) by the Kruskal-Wallis non-parametric ANOVA test. When overall statistical significance was observed (p<0.05), pairwise comparisons were carried out using the Mann-Whitney U-test. Similar statistical analysis was conducted by region (medial, perifornical and lateral) of the HCRT field [42]. The distribution of intensities of *Per1* signal within these cell populations provides information regarding the pattern of clock gene expression within and between functionally interconnected but disparate nuclei and their subfields. To this end, the intensity of *Per1* signal above threshold was assessed for all cells in each area for all animals in each experiment. Next, percentages of cells falling within the highest 25%, middle 50% or lowest 25% *Per1* intensity bins were evaluated for AVP, HCRT, and TH cells of the SCN, DMH/LH and LC respectively at each circadian phase, and differences in this distribution between phases for each cell type were evaluated by nonparametric tests as described above. Within animal assessment of *Per1* expression symmetry was carried out at each brain region of interest, including cell counts and quartile distribution (see next section for description). This provided a reference point for symmetry in DD housed hamsters, which aided in interpretation of the data from the next experiment.

For experiment 2, evaluation of *Per1* expression was carried out in order to assess lateralization between the hemispheres of each brain region. Cell counts and intensity of *Per1* expression were evaluated with Mann-Whitney U-test between the high *Per1* expressing side (High side) versus the low *Per1* expressing side (Low side). As a measure of asymmetry, the mean intensity of *Per1* expression on the high side was divided by the mean *Per1* intensity on the low side and compared to the control group using the Mann-Whitney U-test. The within animal distribution of *Per1* intensity was assessed in two ways. First, the Kolmogorov-Smirnov test was used to determine whether the *Per1* intensity distribution among HCRT cells differed between sides within each animal. Second, the percent of HCRT cells expressing a *Per1* signal that fell in the high, moderate and low bin was determined within each animal and the group mean percentages were compared between the high and low sides using the Mann-Whitney U-test.

In order to determine whether lateralization of *Per1* expression in the LH/DMH and LC was systematically related to asymmetry in the pacemaker, we assessed whether our quantification of the intensity of clock gene mRNA ipsilateral vs. that contralateral differed systematically from that in AVP cells of the SCN of the same animal. If a lateralized timing signal originating in the neurons of the SCN maintains the phase angle of *Per1* expression within the arousal-promoting cells, then a consistent anatomical relationship is expected when assessing the ipsilateral versus contralateral sides of each brain region even though the circadian phase of peak asymmetry may differ between the SCN and the LH/DMH or LC. Spearman’s correlation was calculated in order to determine whether there was a phase-specific relationship between the level of asymmetry observed in the SCN and HCRT cells. Spearman’s correlations were also used to assess the relationship between characteristics of locomotor activity (volume, duration and intensity of the wheel revolutions) and the degree of asymmetry between HCRT fields.

## Results

### Experiment 1: Per1 Expression in Neuronal Cell types of free Running Hamsters

#### AVP cells of the SCN

**Figure 1:**
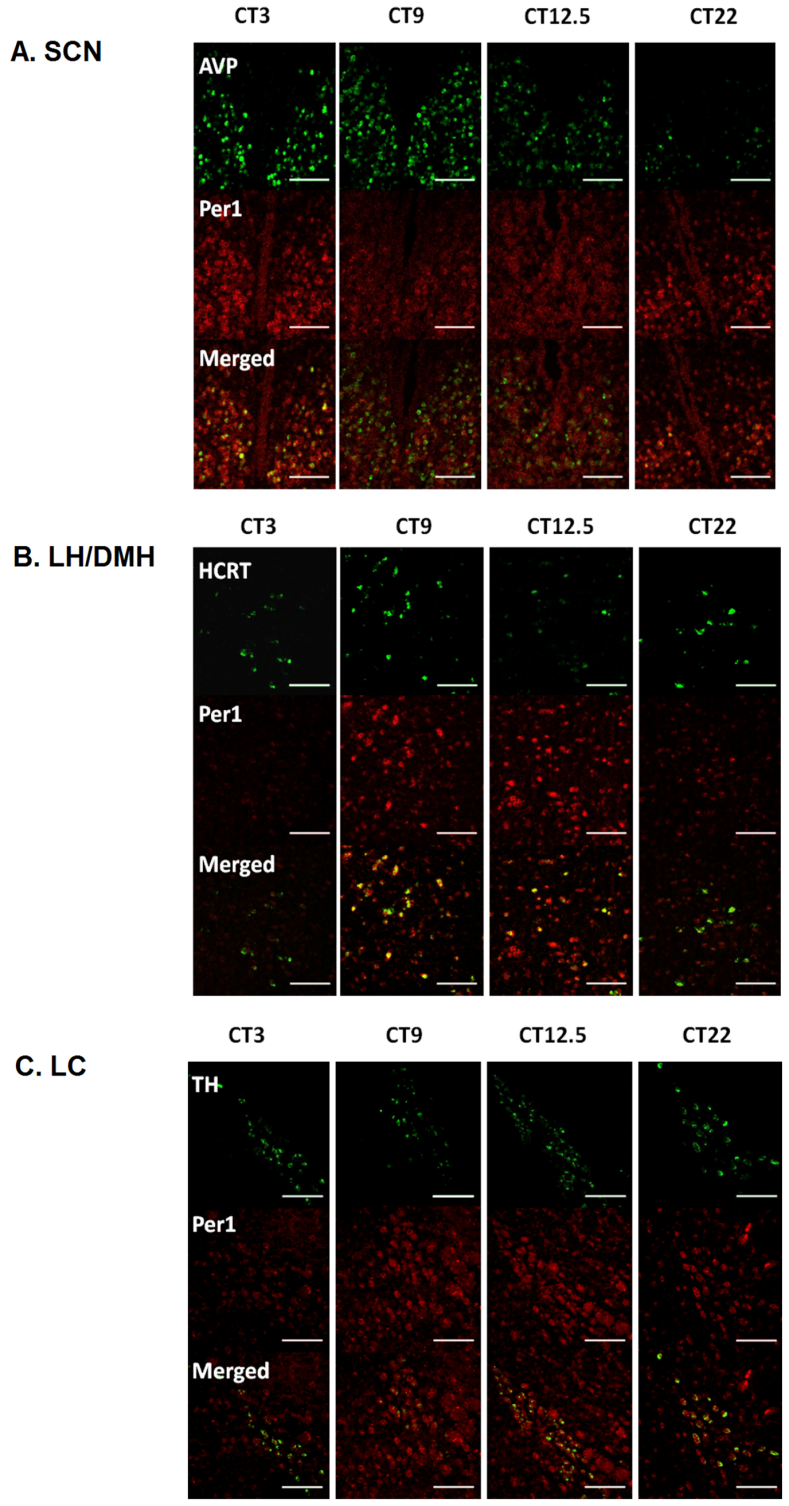
Representative confocal images taken at the level of the (A) SCN, (B) perifornical LH/DMH, (C) LC. Within each brain region, top row shows cells detected in the Alexa488 channel, middle row shows the HNPP/FR signal indicating *Per1* and bottom row is the merged image. A single ∼1micron thick section of a 10 section z-stack was selected as a representative image. Scale bar equals 100 microns. Within each brain region, a section taken from representative animals killed at CT3, CT9, CT12.5 or CT22 is shown.

**Figure 2:**
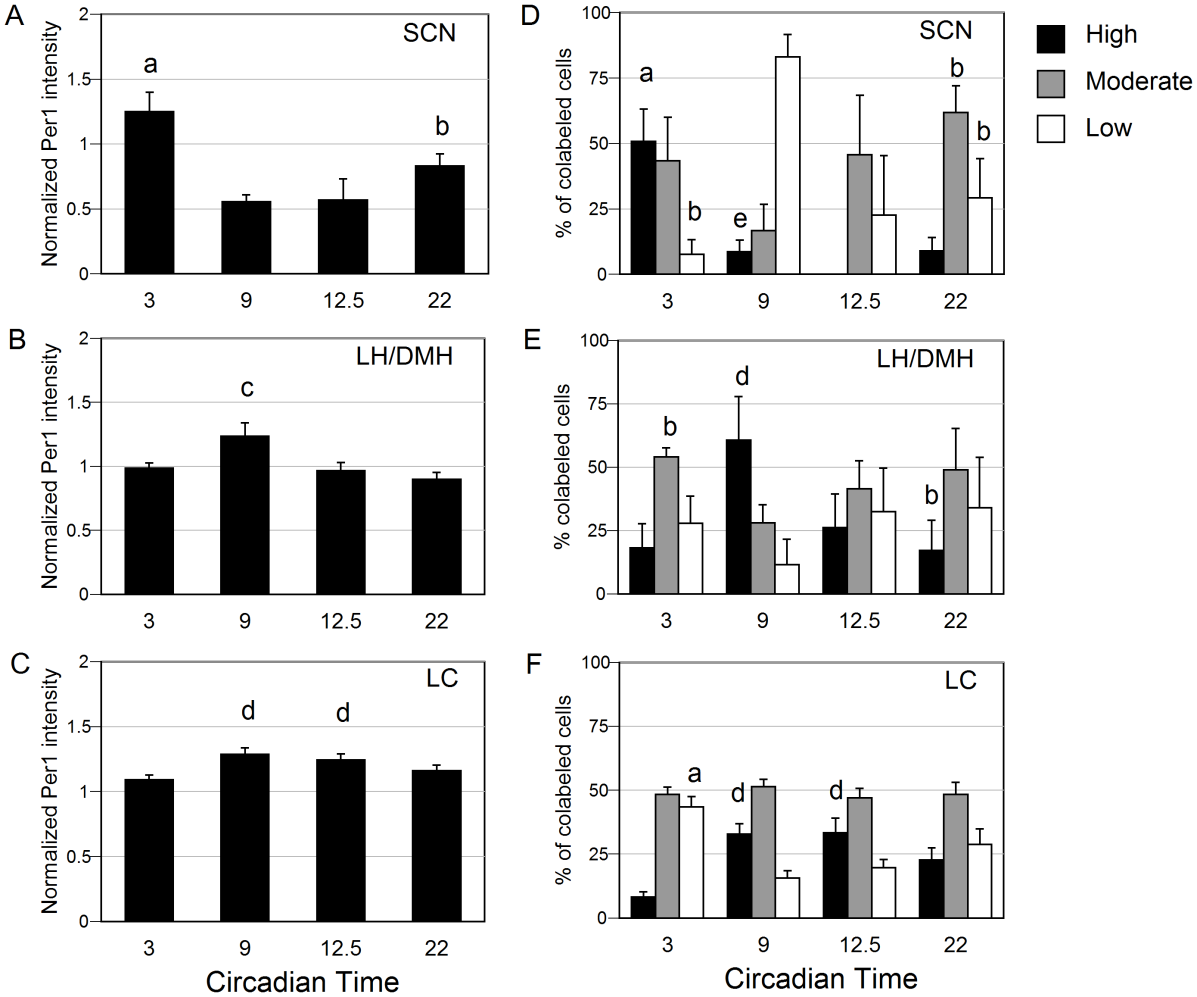
Assessment of *Per1* expression in experiment 1. Left panels show mean colocalization of *Per1*, and right panels indicate distribution of *Per1* expression intensities at each circadian phase, for *AVP*, *HCRT*, and *TH* cells in the brain areas examined. Normalized mean (± SEM) intensity values of *Per1* expression is depicted within (A) *AVP*-expressing cells of the SCN, (B) *HCRT-*expressing cells of the LH/DMH and (C) *TH*-expressing cells of the LC. Percent of cells expressing highest 25% (*black*), moderate (*grey*), or lowest 25% (*white*) levels of *Per1* within (D) *AVP*-expressing cells of the SCN, (E) *HCRT-*expressing cells of the LH/DMH and (F) TH-expressing cells of the LC. a- p<0.05 vs. CT9, 12.5, 22; b- p<0.05 vs. CT9; c- p<0.05 vs. CT3, 12.5; d- p<0.05 vs. CT3; e- p<0.05 vs. 12.5.

Representative images of the middle plane along the rostral-caudal axis of the SCN are presented in figure 1A. As expected, *Per1* expression varied significantly with circadian time. The total number of *AVP* cells counted and the number of *AVP* cells that colabeled with *Per1* were both two-fold higher at CT3 than at CT22 (p<0.05, table S1). The number of colabeled *AVP* and *Per1* cells as well as the percentage of co-labeled *AVP* cells were higher at CT3 than at CT12 (p<0.05; table S1). Of the *AVP* cells colabeled with *Per1*, the normalized *Per1* intensity level at CT3 was significantly greater than at all other phases (p<0.5; figure 2A). The range of *Per1* signal intensities of all cells in the experiment was divided into three parts, and the distribution of high, moderate and low expressing cells was quantified for each circadian phase as a percentage of cells counted. The percentage of *Per1/AVP* colabeled cells that expressed high levels of *Per1* was greater at CT3 than at each of the other phases (p<0.05; figure 2D).

We assessed the symmetry of *Per1* expression in the middle plane of the SCN of hamsters maintained in DD. Of the colabeled cells, the *Per1* intensity level was similar between the sides of the SCN and the high to low ratio of *Per1* expression did not differ between phases (figure S4A and S4D).

#### HCRT cells of the LH/DMH

Representative images of the perifornical HCRT field from animals killed at CT3, 9, 12.5 or 22 are included figure 1B. Of the phases sampled, *Per1* expression was higher at CT9 than at CT3 and CT12.5 (*P*<0.05; figure 2B). A similar number and percentage of HCRT cells met the Manders’ coefficient (m≥0.5) of colocalization across the phases sampled (table S1). In order to assess regional distribution of *Per1* expression within the hypocretin field the medial, perifornical and lateral regions were assessed separately. A similar expression pattern of *Per1* was observed in the medial and perifornical regions. In contrast, *Per1* intensity did not differ with phase in the lateral region (p>0.05; figure S3). *Per1* intensity within *HCRT* fields was similar on both sides of the brain of DD hamsters, so that the high to low ratio was close to 1.0 at all phases analyzed (figure S4B and S4E). The percentage of Per1/HCRT colabeled cells that expressed high levels of *Per1* was greater at CT9 than CT3 (P<0.05; figure 2E).

#### TH Cells of the LC

Representative images of the rostral LC are shown for each phase sampled (figure 1C). A similar number of TH-expressing cells and percentage of *TH*-expressing cells showed *Per1* signal above background at each circadian time (table S1). Nevertheless, the mean level of *Per1* expression in TH cells was higher at CT9 and CT12.5 than at CT3 (p<0.05, figure 2C). *Per1* labeling in TH cells was bilaterally symmetrical at each phase (figure S4C and S4F). The percentage of *Per1/TH* double-labeled cells that expressed high levels of *Per1* was greater at CT9 and CT12.5 than CT3 (P<0.05; figure 2F).

### Experiment 2: Per1 Expression in Neuronal Cell types of Split Hamsters

#### AVP cells of the SCN

**Figure 3:**
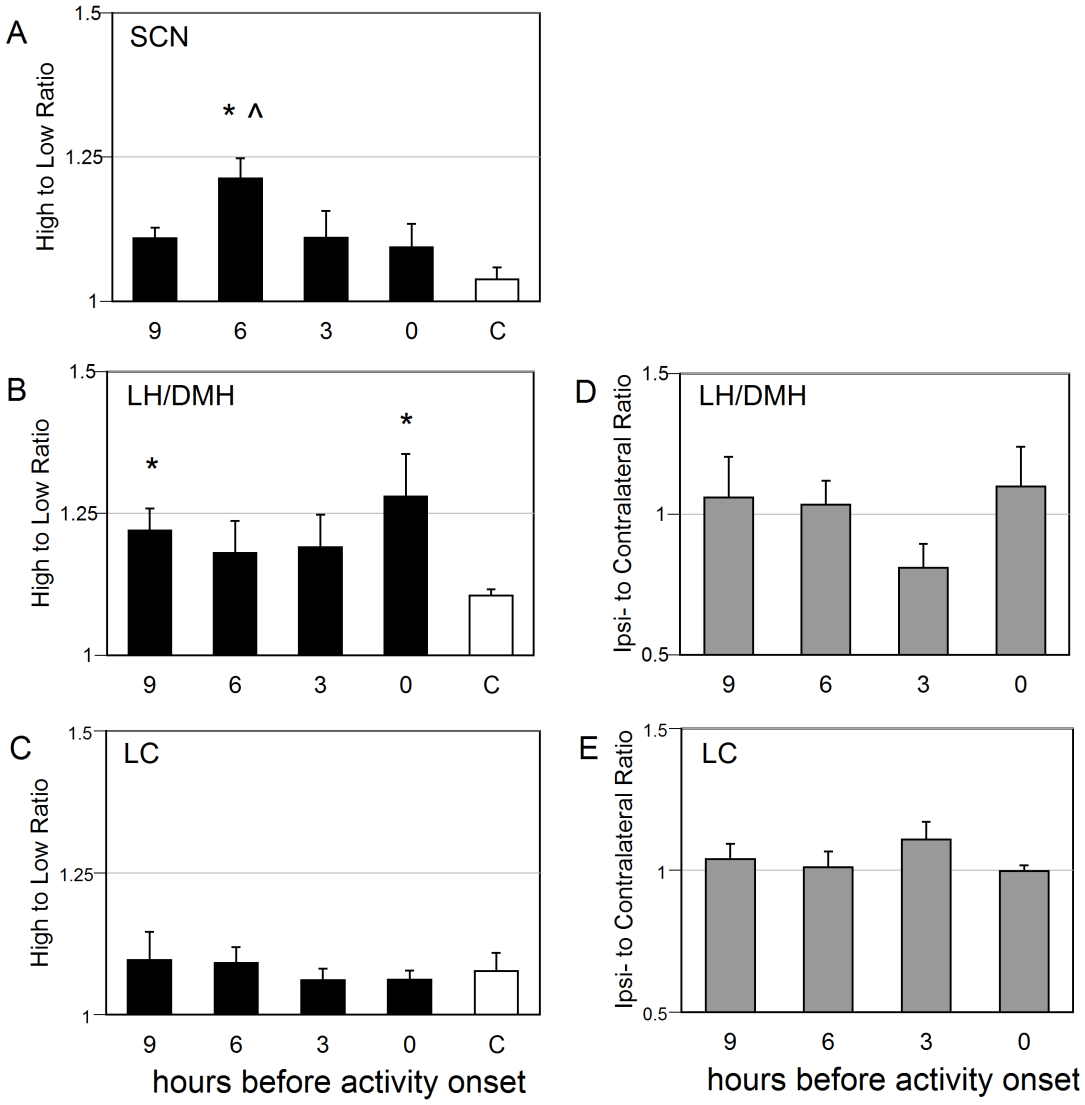
Asymmetry in the mean (±SEM) intensity of *Per1* expression among hamsters maintained in LL in experiment 2. *Per1* expression was lateralized in cases in which the high to low ratio of *Per1* expression exceeded that in non-split controls. The high to low ratio of *Per1* expression is illustrated in the (A) *AVP*-expressing cells of the SCN, (B) *HCRT-*expressing cells of the LH/DMH and (C) *TH*-expressing cells of the LC. Consistency of lateralization to the high *Per1* expressing SCN was assessed by the ipsilateral to contralateral ratios of *Per1* expression in (D) *HCRT-*expressing cells of the LH/DMH and (E) *TH*-expressing cells of the LC. * p<0.05 vs. C; χ p<0.05 vs. 9 h before activity onset.

**Figure 4:**
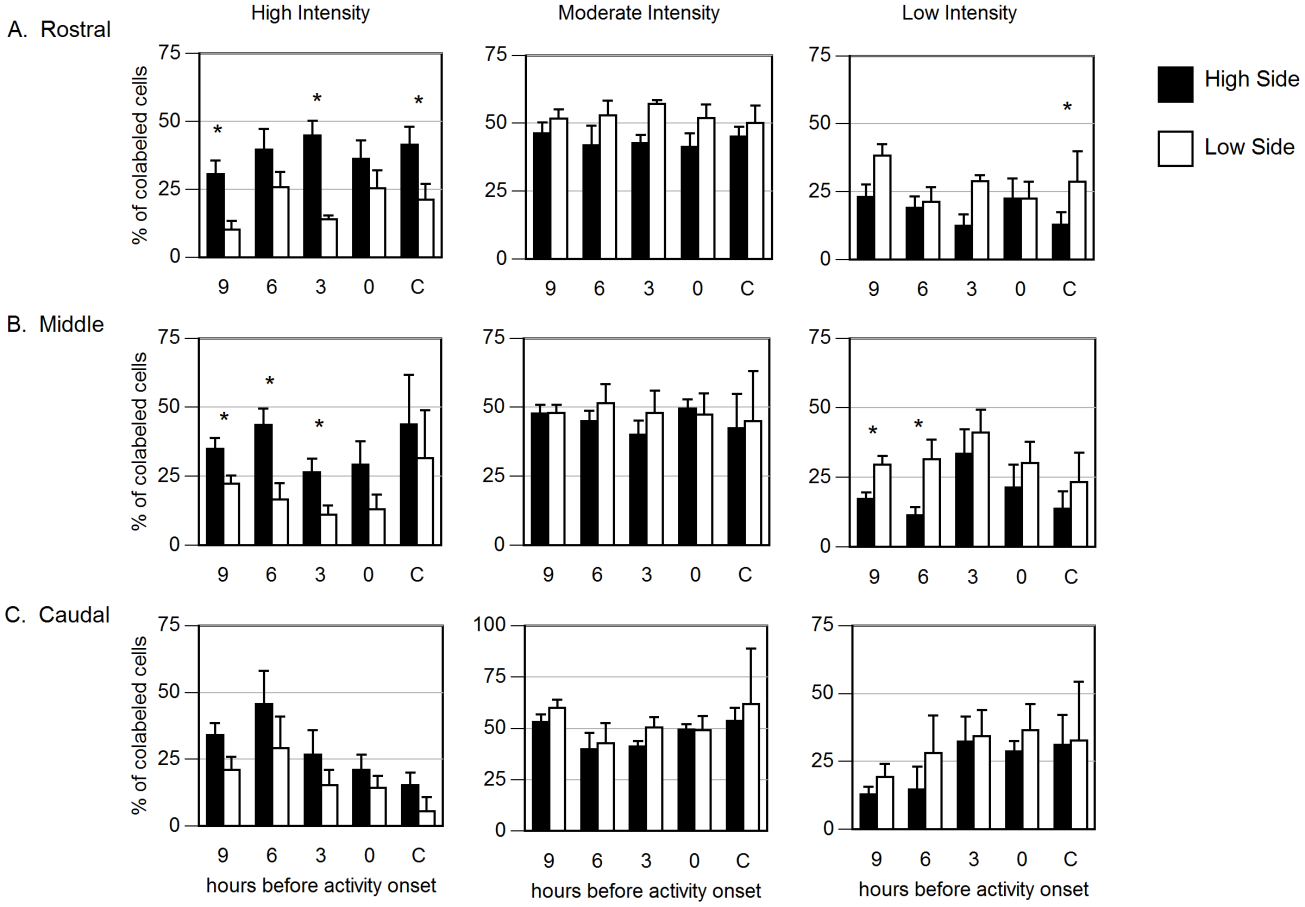
Assessment of the distribution of *Per1* expression intensities in the SCN of hamsters exposed to LL. Distribution of cells expressing high (*left*), moderate (middle two quartiles; *center*) or low (*right*) intensity of *Per1* hybridization in (A) Rostral, (B) Middle or (C) Caudal SCN. * p<0.05 vs. low side.

The number of cells expressing *AVP* on each side of the SCN, and the number of *AVP* cells that also expressed *Per1,* were similar across circadian phases in each plane (rostral, middle and caudal) of the SCN (see table S2). The number of *Per1* cells was also similar between the sides of the SCN. Consistent with earlier reports, we found that LL exposure adequate to induce splitting of locomotor activity resulted in regionally and phenotypically specific asymmetry of *Per1* expression in the SCN: within *AVP* cells colabeled with *Per1* in the middle plane of the SCN, the ratio of high to low *Per1* intensity was greater at 6 h before AO than at 9 h before AO (p<0.05; figure 3A) and greater than unsplit control (p<0.05). Of the *AVP* cells co-labeled with *Per1*, the percent in the highest quartile of *Per1* expression differed between the coronal planes of the SCN. In the rostral SCN, significant asymmetry was evident at 9 h and 3 h before AO in (p<0.05 vs. low side; figure 4A). In the middle SCN, asymmetry occurred at 9 h, 6 h, and 3 h before AO (figure 4B). No asymmetry was seen at any phase in the caudal SCN (figure 4C).

#### HCRT-cells in the LH/DMH

**Figure 5:**
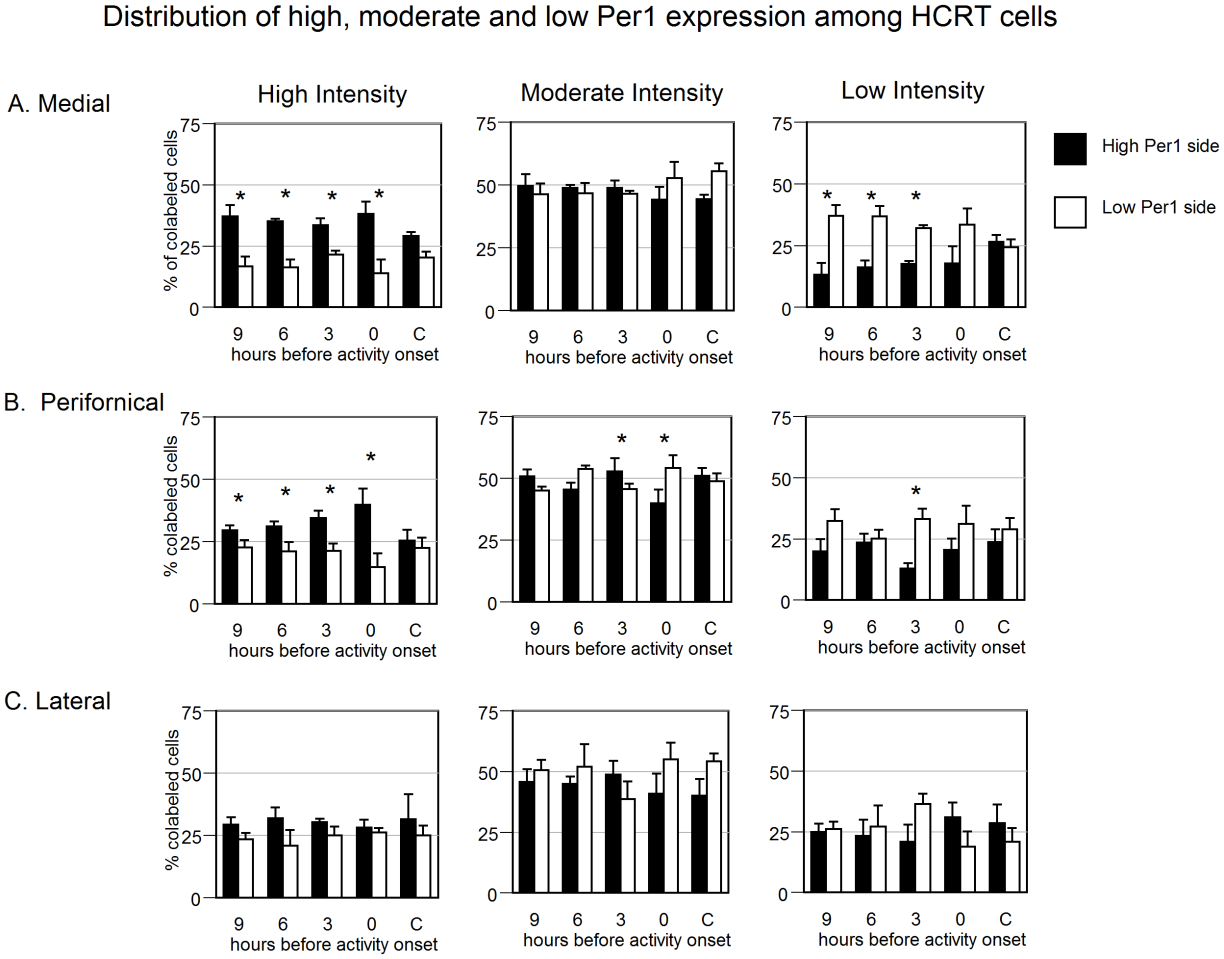
*Per1* intensity quantified by category of intensity within HCRT cells. Distribution of cells expressing high (*left*), moderate (*center*) or low (*right*) levels of *Per1* in the (A) medial, (B) perifornical and (C) lateral regions of the *HCRT*-expressing cells of the LH/DMH. * p<0.05 vs. low side.

The total number of *HCRT* cells counted per side of the LH/DMH and the number of *HCRT* cells that expressed *Per1* above the threshold was similar at all circadian phases (see table S2). In split hamsters, the asymmetry of *Per1* labeling in HCRT cells exceeded that in unsplit controls at 9 h before AO and at activity onset (p<0.05; figure 3B). When evaluated by region, asymmetry of *Per1* levels exceeded that in unsplit controls at 9 h before AO and at activity onset in the medial region (p<0.05; figure S5) and approached significance in the perifornical region at 9 h pAO versus unsplit controls (p = 0.07). The lateral region was symmetric at all phases. The percent of HCRT cells showing the highest quartile of *Per1* expression was asymmetric at all phases assessed in split hamsters, but symmetric within unsplit controls. When assessed by region, the medial and perifornical HCRT field had significant lateralization in the percentage of high expressing *Per1* cells (figure 5), but the lateral HCRT field did not (figure 5).

*Per1* intensity levels within HCRT cells on the side ipsilateral vs. contralateral to the high *Per1* expressing SCN (I:C ratio) differed at 9 h before AO in 4 of 5 animals (p<0.05, Kolmogorov-Smirnov test) whereas lateralization was inconsistent at the other phases sampled (occuring in only 1 of 5 animals at 3 h and 6 h before AO and in 2 of 5 hamsters sampled at AO). The I:C ratio of *Per1* expression tended to be lower at 3 h vs. 0 h before activity onset in HCRT cells (p = 0.08; figure 3D). We sought to assess further the relationship of lateralization of *Per1* expression in HCRT cells to asymmetry of circadian function in the SCN. Thus the relationship between the level of asymmetry in *AVP*- expressing cells of the middle SCN and the asymmetry of I:C or H:L ratios of *Per1* expression in HCRT cells of the medial and perifornical regions were assessed using Spearman’s correlation. A significant positive correlation between the medial and perifornical HCRT I:C ratio with the H:L SCN ratio was observed at AO (figure S6).

No significant correlation between behavioral rhythms (locomotor activity bout volume, intensity or duration) and asymmetry of *Per1* expression within HCRT cells was observed (Spearman correlation p>0.05 for each correlation analysis).

#### TH cells in the LC

**Figure 6:**
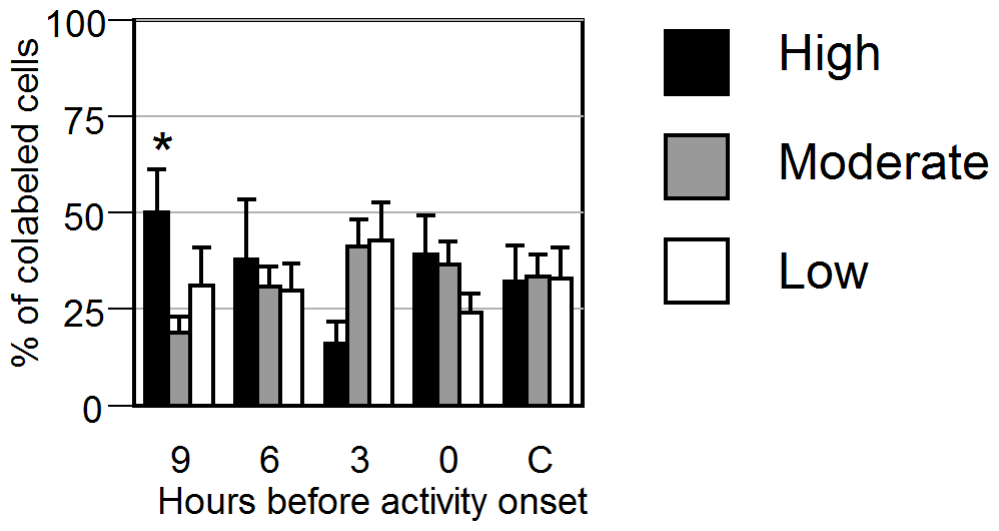
*Per1* intensity quantified by category of intensity within TH cells. Distribution of cells expressing high (*black*), moderate (*grey*) or low (*white*) levels of *Per1* in the *TH*-expressing cells of the LC. * P<0.05 vs. 3 hours before activity onset.

The total number of *TH* cells counted per side of the LC and the number of *TH* cells that expressed *Per1* was similar, as was the percentage of co-labeled *TH* cells (see table S2). There was no evidence of lateralization of *Per1* expression in *TH* cells of split hamsters: the high to low ratio and the ipsilateral to contralateral ratios were close to 1.0 at all phases (figure 3C and 3E). As the ratios indicate a symmetric relationship between the sides of the LC, the data were collapsed and the distribution of *Per1* expression across phases was assessed. Nevertheless, the percentage of cells in the highest quartile of *Per1* expression was greater at 9 h than at 3 h before AO (p<0.05; figure 6).

## Discussion

### Clock gene Expression within Arousal-Promoting Neurons

Core clock genes are expressed in multiple cell types in mice, rats and hamsters [44]–[49]. Within the brain, clock gene expression also appears to be widespread [2], [26], [28]–[30], [50]–[54]. The results of the current study indicate that the clock gene, *per1*, is expressed in HCRT cells of the LH/DMH and *TH* neurons of LC. Furthermore, the message levels of *Per1* change across the day. As in the case of peripheral oscillators, the phase of the oscillation of *Per1* expression in these arousal-regulating neurons lags that of the master pacemaker.

Our findings are consistent with earlier observations that the expression of *Per1* within the SCN is high in the subjective day and low during the subjective night in hamsters [44], [54]–[59] as in other rodents [60]–[62], with peak expression around CT4 and a nadir at approximately CT18. We have expanded observations of regionally specific rhythmic *Per1* expression in the SCN shell [57] to cellular co-expression in *AVP* neurons. The amplitude of the rhythms of clock gene expression detected through this dual label *in situ* hybridization method is lower than that found using isotopically labeled probes or immunostaining. This may reflect the focus on specific cell types that we were able to achieve using this method, or the fact that in the isotopic method the signal is generated from cells at multiple levels of the section so that stereological considerations contribute to the apparent amplitude. Additional technical issues, including the linearity of signal amplification of both the *Per1* and the phenotypic signal, also make it difficult to compare directly the amplitude of the rhythm we examined with that inferred from other methods. Our main purpose in experiment 1 was to assess clock gene expression in particular cell types of two arousal-promoting nuclei that are likely under pacemaker control, and to determine its phase relationship to the SCN. The phase angle difference between clock gene expression in the SCN and other brain regions depends on the area and cell phenotypes investigated [29], [31], [63]; current results]. Peak clock gene expression in peripheral organs typically lags the master pacemaker by several hours [48], [64]. Similarly, peak reporter luminescence in explants of *Per1-luc* rats and PER2::LUC mice occurs around subjective dusk in the SCN region and in the mid-subjective night in the arcuate area, the lateral hypothalamus and paraventricular hypothalamic region [63], [65]. Limitations of sampling frequency restrict our ability to define with precision the phase of peak clock gene expression within the arousal-promoting cells. Nevertheless, our findings demonstrate a lag in the phase of *Per1* expression between the vasopressinergic SCN and both *HCRT*- and *TH*-expressing cells.

Multisynaptic relays between the pacemaker and subordinate oscillators may account at least in part for the latency with which *Period* gene expression peaks. The SCN may regulate DMH function by either direct [66]–[68] projections or by relays through the sub-paraventricular zone [69], [70]. In either case, there is clear anatomical evidence for SCN regulation of HCRT cell activation [23]. Moreover, the *AVP*-expressing cells of the SCN project more densely to the DMH than do the more ventrally situated VIP cells [66]. Yoshida et al. [68] observed that SCN neurons innervate the HCRT field medial to the fornix. The multiplicity of regional inputs to the HCRT field and heterogeneity of LH/DMH cell populations [71], [72] and activation patterns [73], [74] suggest that both direct and indirect SCN inputs may regulate *Per1* expression in this region.

Although vasopressinergic outputs may serve as important relays [75], other SCN efferents whose peak phases may differ may also regulate subordinate oscillators including the DMH/LH and LC [65], [67], [76]. It is also not clear which components of the circadian mechanism are most directly responsive to SCN outputs. While the initial step in the entrainment of the SCN pacemaker by light and locomotor activity is transcriptional activation or suppression of *Per1* [55], [60], [77], *zeitgebers* that shift the phase of subordinate oscillations may more directly regulate expression of other clock genes [78]–[80]. Further investigation of the entrainment mechanisms of subordinate oscillators in brain areas including the LH/DMH and LC is needed.

The amplitude of *Per1* rhythms was higher in the *AVP* cells of the SCN than either *HCRT* neurons of the LH/DMH or *TH* neurons of the LC. We found HCRT cells that expressed *Per1* at low levels at each phase sampled. We cannot discriminate between the possibility that *Per1* expression oscillates only in a subpopulation of *HCRT* cells of the LH/DMH, or *TH* cells of the LC, or that most or all of these cells oscillate but peak at different phases. Such phase clusters occur within the SCN [81]–[84] and it is possible that SCN heterogeneity is conveyed to different cell populations in LH/DMH, LC, or other brain regions. We may have missed the peak or nadir of the *Per1* oscillation in any or all of these areas due to the limitations of sampling frequency. On the other hand, we find a more pronounced oscillation than is suggested by the results of Salgado-Delgado et al. [31], who found no rhythm of PER1 immunoreactivity within the LH/DMH region of rats but reported that immunoreactive cell numbers increased when locomotor activity was induced between ZT2 and ZT10. Our examination of phenotype-specific *Per1* expression may have enabled resolution of clock function that is not general to all cells in this region. Within the DMH/LH, HCRT and MCH cells have opposing functions that likely result in different phases of activation, but it remains to be determined whether clock gene expression is asynchronous [85]–[89]. A limitation of *in situ* hybridization or immuncytochemical studies is that they capture only one phase per animal. Use of a reporter gene driven by a clock gene promoter may allow for assessment of the HCRT population, *in vivo* or *in vitro,* in real time, provided that methodological hurdles to identification of cell phenotype along with clock gene expression can be surmounted [11], [90]. Use of tract-tracing approaches with phenotype labeling and phase markers may identify precisely which subpopulations of HCRT cells participate in a particular behavioral and/or physiological circuit and which possess a molecular clock.

### Lateralized SCN Communication to Arousal-Promoting Neurons

Consistent with immunocytochemical studies, asymmetry of *Per1* expression was evident in the rostral and middle regions of the SCN [62], [91], [92]. Yan et al. [91] proposed that the split state is marked by synchrony between the rostral and contralateral middle region of the SCN. In the present study, we observed one animal at each phase in which the side expressing high *Per1* differed between the rostral and the middle SCN. Although a distributional analysis of *Per1* expression provided a less stringent cutoff, the rostral and middle planes differed in less than half of the hamsters (6 out of 14 animals killed at 9 h, 6 h or 3 h before activity onset). These observations suggest that multiple phase relationships may occur among groups of SCN neurons in hamsters induced to split by exposure to constant light.

In some instances, diffusible signals may relay phase information from the SCN [36]. In others, blood-borne or temperature and/or food cues that mediate pacemaker influences on the phase of peripheral oscillators may regulate clock gene expression in the brain [4], [37], [46], [93] In each such case, clock function on the left and right sides of the brain would be expected to be symmetrical. Our intent was to use the split preparation as a tool to examine SCN control of efferent targets that might act as subordinate oscillators. Efforts to assess the relationship between the SCN and the LH/DMH and LC are complicated by the phase differences of peak *Per1* expression between areas. Thus it is difficult to assess pacemaker control of clock gene expression in its efferent targets in a single animal, as transcription in the LH/DMH or LC at the time of sacrifice may have been controlled by events that occurred in the SCN many hours previously. The level of asymmetry within the HCRT field appeared to peak during the last few hours before activity onset, about 6 hours after the peak in asymmetry in the middle SCN. A similar lag was observed between peak *Per1* in the SCN and HCRT cells of DD-housed hamsters. At 3 h before activity onset, the high *Per1* expressing HCRT cells tended to be on the side contralateral to the high *Per1* expressing SCN. At activity onset the opposite relationship exists. HCRT cells likely oscillate in antiphase in split hamsters, as is the case for AVP cells of the SCN. However, non-lateralized cues including diffusible SCN substances, humoral, temperature, or other activity-dependent cues available to both sides may diminish the extent of lateralization of clock gene expression [31], [36], [94]. The oscillation in free running hamsters of *Per1* expression in *TH* cells of the LC, while statistically significant, was of lower amplitude than that in *AVP* cells of the SCN or *HCRT* cells of the LH/DMH. This may have made it difficult to detect lateralization in LL-exposed hamsters that split in experiment 2. Sampling at other phases relative to activity onset may be necessary to detect lateralization of clock gene expression in the LC.

Recently, Butler et al. [95] observed bimodal co-expression of FOS-ir within HCRT cells of hamsters induced to split by exposure to LL. They quantified co-expression in a region centered on the portion of lateral hypothalamus in which HCRT cells were most highly concentrated. Thus their analysis included a region lateral to that in which we found the greatest evidence of rhythmic *Per1* expression in experiment 1. Butler et al. [95] observed a high amplitude fluctuation between the percentage of activated HCRT cells in split animals, but found no significant lateralization of HCRT/FOS co-labeling. Although they interpreted FOS expression to reflect entrainment of an efferent target of the SCN, they did not observe a measure of the phase of a circadian oscillator within the HCRT cells. In contrast, we assessed mRNA encoding a clock gene whose expression may not always directly correspond to cell activation [96], [97]. Different patterns of SCN input, or co-release of signals, may control activation vs. circadian phase, and the pattern of FOS/PER1 co-expression characteristic of retinorecipient cells of the SCN may not also be characteristic of SCN targets. This could be the case if output signals of the SCN responsible for entrainment of subordinate oscillators do not lead to activation of common promoter elements in clock and immediate early genes. Furthermore, FOS expression in the DMH/LH may be more readily influenced by feedback cues (including temperature and feeding) associated with the split behavior than is the transcription of clock genes. Beyond the differences between our study and that of Butler et al. [95] in the genes whose expression was assessed and the examination of protein vs. mRNA, we used a distributional analysis of message level rather than an all-or-none categorization of whether cells are activated or not.

Butler et al. [95] estimated a 6-hour lag between the phase of peak cell activation in the SCN shell and the HCRT cells. Peak FOS-ir within the SCN shell of behaviorally split hamsters occurred at approximately 3 hours before activity onset, whereas peak FOS within HCRT cells occurred at 3 hours after activity onset. This 6-hour lag is consistent with the lag we observe in *Per1* expression within the AVP cells of the SCN and the HCRT cells of the LH/DMH. We observed a rhythm of *Per1* expression in the medial and perifornical but not the lateral population of HCRT cells. This is interesting given the observations that HCRT cells in these subregions differ in projections and presumptive functions [70], [85], [98], [99] The numerous interactions (both efferent and afferent) between the HCRT field and other regions suggest that splitting of HCRT function may have significant behavioral and physiological impact.

In contrast to *Per1* expression in the LH/DMH, the LC of behaviorally split hamsters remained symmetric, although the limited phases sampled may have prevented detection of lateralization at the level of the LC. Furthermore, the nadir of *Per1* expression within *TH*-expressing cells of the LC occurred at about 3 hours before activity onset, suggesting that the phase relationship between the LC and activity onset in behaviorally split hamsters differs from that of DD-housed hamsters. Projections from the hypocretin field to the LC may be bilateral, therefore reducing the lateralized signal from the SCN to distant regions and originate across the HCRT field. Diffusible outputs of the SCN or even the DMH may regulate the circadian phase of the LC [14], [69], [70], [99]-[107], and this may contribute to lack of lateralization of *Per1* expression. The effects of systemic humoral signals including adrenocortical hormones may also account in part for symmetry of clock function in the LC [108] as elsewhere in the brain [2]. Locomotor feedback may also reach the LC, preventing lateralized top-down signals from entraining clock gene expression in these cells [109], [110]. It remains an open question whether completely crossed or uncrossed cues from the SCN might regulate *Per1* expression in the LC, but global and/or bilateral cues cannot be ruled out.

### Conclusion

We have documented time-dependent changes in the expression of the core clock gene *Per1* in arousal-promoting cells, and found that the phase of peak *Per1* expression in these cells lags that observed in the SCN. We also observed regional lateralization of clock gene expression at specific phases between the sides of the medial *HCRT* population that is consistently related to that in *AVP* cells of the SCN. Lateralization of clock gene expression was not evident in *TH*-expressing cells of the LC. Identification of the transmitter(s) that control circadian phase will help elucidate integrative properties of timing cues. Investigation of the function of a molecular clock within specific neuronal populations will clarify the circadian modulation of behavior and physiology.

## Supporting Information

Figure S1 **Representative actograms of hamsters used in these experiments.** (A) Locomotor activity record of an animal placed in constant darkness (DD) for ten days. Asterisk indicates kill time at CT12.5 for Experiment 1. (B) Record of a hamster maintained in LL for Experiment 2. Although only the 33 days leading up to the kill time are shown here, the morning and evening components can be identified by tracing the time of activity onset to the record preceeding the split (see figure 1 in Mahoney et al., 2010). This animal showed a typical split, and was killed at AO (asterisk).
(TIF)

Figure S2 **HCRT expression in the Syrian hamster brain.** Autoradiogram illustrating regional distribution of *HCRT* in the Syrian hamster as determined using 35S-labeled probe. Scale bar is 1 mm.
(TIF)

Figure S3 **Expression of** ***Per1*** **in subregions of the HCRT field.** Normalized mean (±SEM) intensity values of *Per1* expression within the medial (*black*), perifornical (*gray*) and lateral (*white*) regions of the hypocretin field of DD-housed hamsters. a- p<0.05 vs. CT, 12.5, 22.
(TIF)

Figure S4 **Symmetry of** ***Per1*** **expression within DD housed hamsters.** Left panels show normalized *Per1* intensity for the high (*black*) and low (*white*) expressing (A) *AVP* cells of the SCN, (B) *HCRT* cells of the LH/DMH and (C) *TH* cells of the LC. The right panels show the high to low ratio of *Per1* expressionin in (D) *AVP* cells of the SCN, (E) *HCRT* cells of the LH/DMH and (F) *TH* cells of the LC. Mann Whitney U analysis indicate a lack of asymmetry in each cell type (p>0.05 between sides in A, B and C) and no effect of phase (p>0.05 between CT points in D, E and F). This analysis allows assessment of effects of LL to induce asymmetry in split hamsters in experiment 2.
(TIF)

Figure S5 **Asymmetry of** ***Per1*** **expression within subregions of the HCRT field.** The high to low ratio of normalized *Per1* intensity in the medial (*black*), perifornical (*gray*) and lateral (*white*) regions of the HCRT field. * p<0.05 vs. unsplit Controls; # p<0.05 vs. 3 h before AO.
(TIF)

Figure S6 **Relationship between asymmetry of** ***Per1*** **expression within subregions of the HCRT field and within** ***AVP*** **cells of the SCN.** Asymmetry of *Per1* expression within the medial HCRT field (A) plotted as the H:L ratios (top) and the I:C ratios of *Per1* expression (bottom) versus the HL ratios of *Per1* expression in *AVP* cells in the SCN at 9 h, 6 h, 3 h or 0 h before AO. Similar plots of asymmetry of the intermediate HCRT field are presented in (B). The *p* and *rho* values are indicated within each plot.
(TIF)

Table S1 **Cell counts for Experiment 1.**
(DOCX)

Table S2 **Cell counts for Experiment 2.**
(DOCX)
