# Supplementary figures and images for: Central Control of Circadian Phase in Arousal-Promoting Neurons
Source: PLoS One. 2013 Jun 24;8(6):e67173. doi: 10.1371/journal.pone.0067173 (PMC3691112; doi:10.1371/journal.pone.0067173)

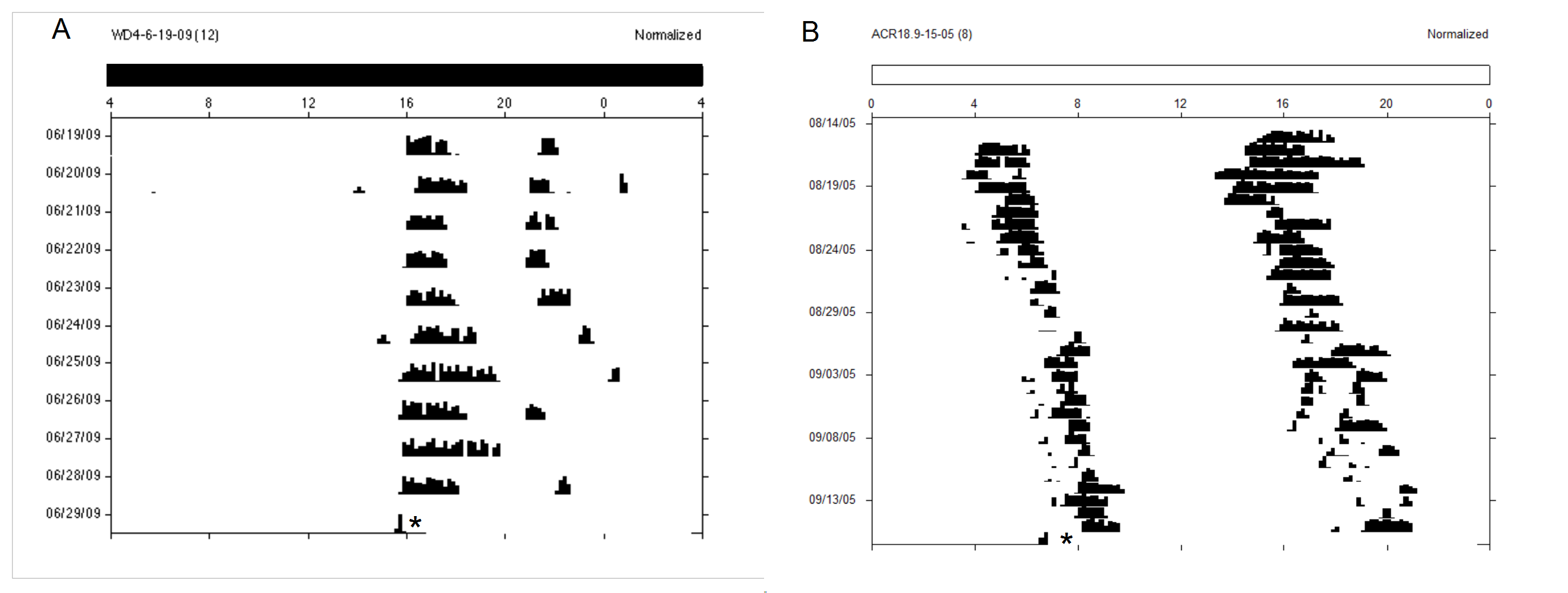

Supplement: Figure S1 — Representative actograms of hamsters used in these experiments. (A) Locomotor activity record of an animal placed in constant darkness (DD) for ten days. Asterisk indicates kill time at CT12.5 for Experiment 1. (B) Record of a hamster maintained in LL for Experiment 2. Although only the 33 days leading up to the kill time are shown here, the morning and evening components can be identified by tracing the time of activity onset to the record preceeding the split (see figure 1 in Mahoney et al., 2010). This animal showed a typical split, and was killed at AO (asterisk). (TIF) [file pone.0067173.s001.tif]

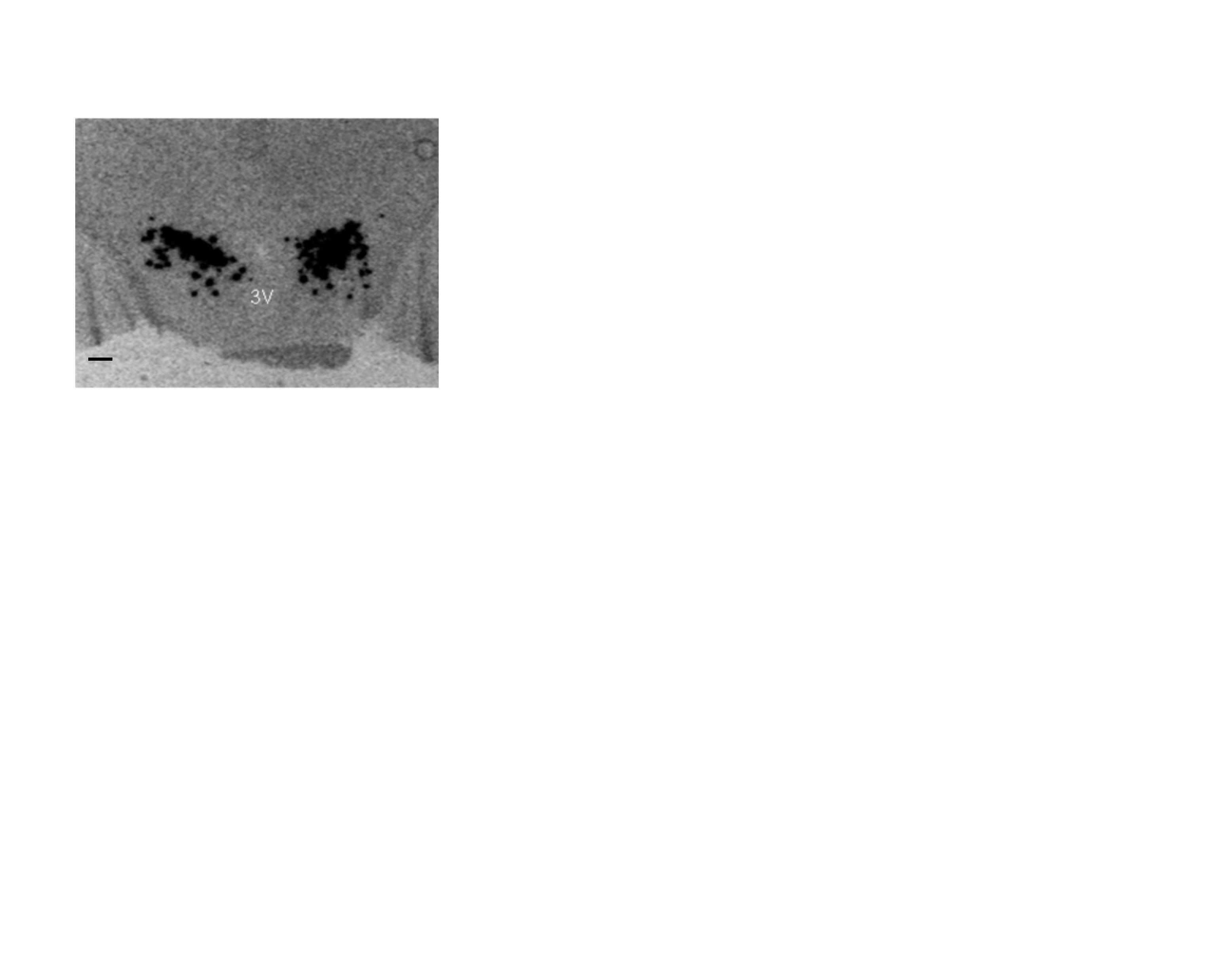

Supplement: Figure S2 — HCRT expression in the Syrian hamster brain. Autoradiogram illustrating regional distribution of HCRT in the Syrian hamster as determined using 35S-labeled probe. Scale bar is 1 mm. (TIF) [file pone.0067173.s002.tif]

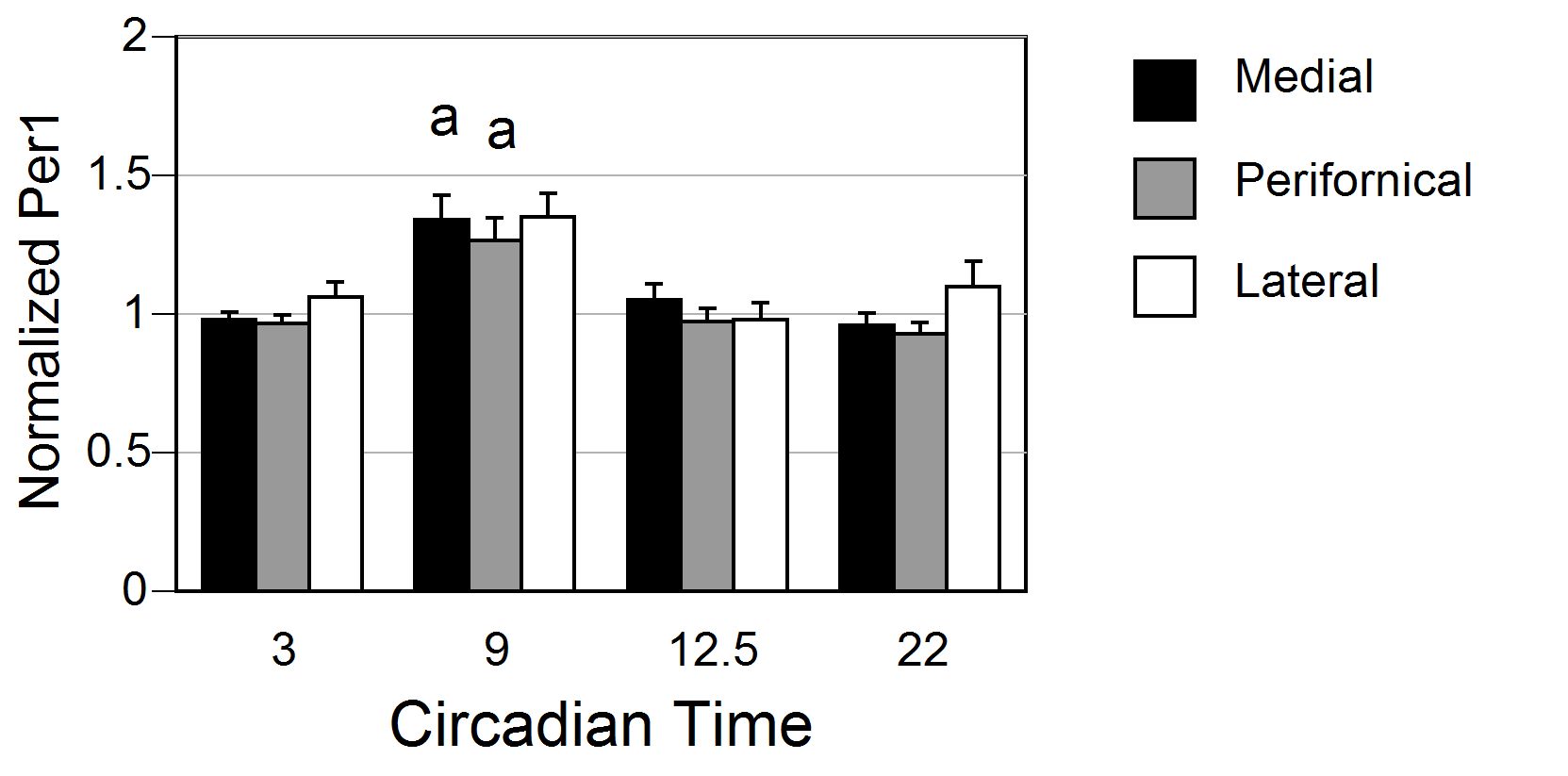

Supplement: Figure S3 — Expression of Per1 in subregions of the HCRT field. Normalized mean (±SEM) intensity values of Per1 expression within the medial (black), perifornical (gray) and lateral (white) regions of the hypocretin field of DD-housed hamsters. a- p<0.05 vs. CT, 12.5, 22. (TIF) [file pone.0067173.s003.tif]

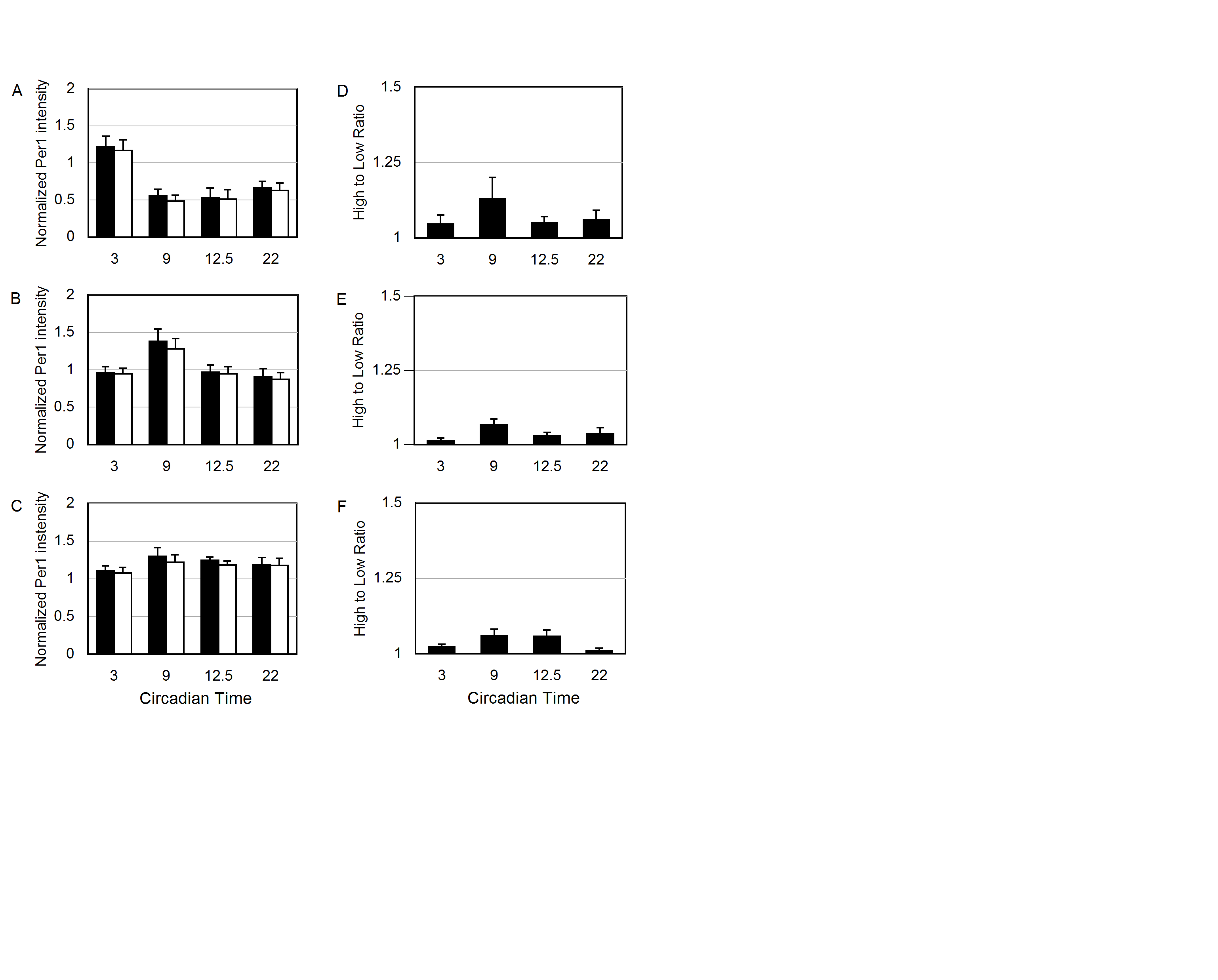

Supplement: Figure S4 — Symmetry of Per1 expression within DD housed hamsters. Left panels show normalized Per1 intensity for the high (black) and low (white) expressing (A) AVP cells of the SCN, (B) HCRT cells of the LH/DMH and (C) TH cells of the LC. The right panels show the high to low ratio of Per1 expressionin in (D) AVP cells of the SCN, (E) HCRT cells of the LH/DMH and (F) TH cells of the LC. Mann Whitney U analysis indicate a lack of asymmetry in each cell type (p>0.05 between sides in A, B and C) and no effect of phase (p>0.05 between CT points in D, E and F). This analysis allows assessment of effects of LL to induce asymmetry in split hamsters in experiment 2. (TIF) [file pone.0067173.s004.tif]

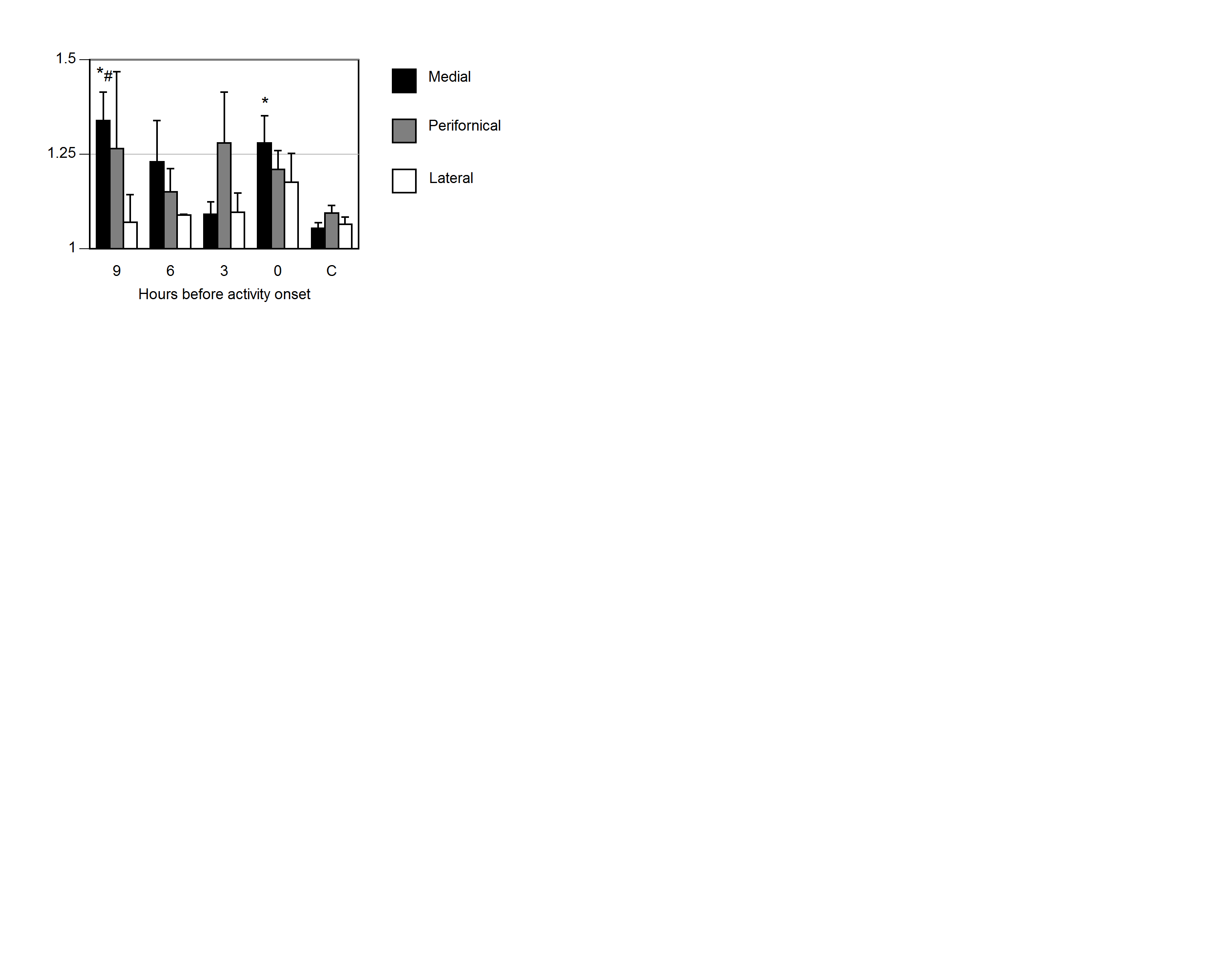

Supplement: Figure S5 — Asymmetry of Per1 expression within subregions of the HCRT field. The high to low ratio of normalized Per1 intensity in the medial (black), perifornical (gray) and lateral (white) regions of the HCRT field. * p<0.05 vs. unsplit Controls; # p<0.05 vs. 3 h before AO. (TIF) [file pone.0067173.s005.tif]

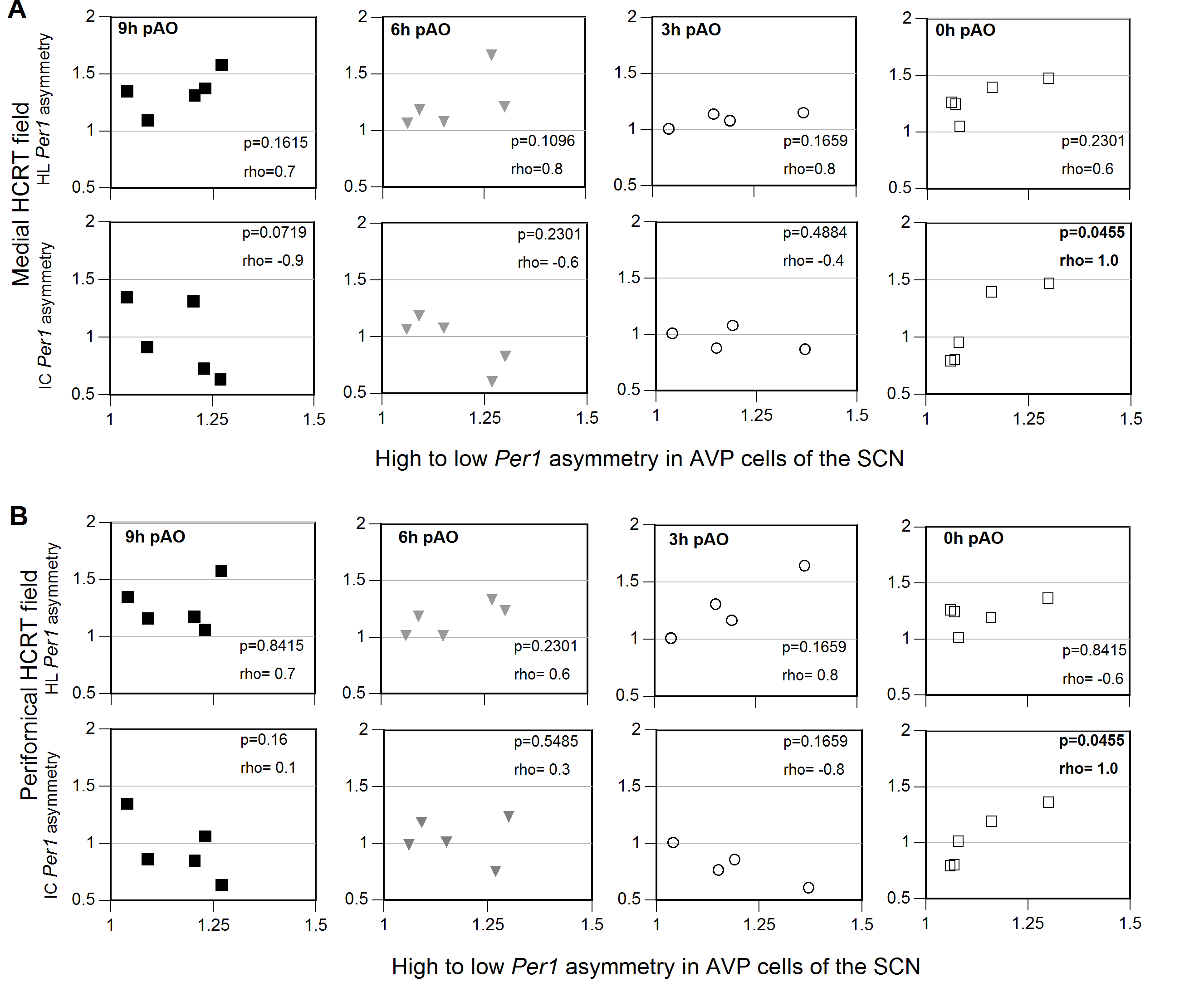

Supplement: Figure S6 — Relationship between asymmetry of Per1 expression within subregions of the HCRT field and within AVP cells of the SCN. Asymmetry of Per1 expression within the medial HCRT field (A) plotted as the H:L ratios (top) and the I:C ratios of Per1 expression (bottom) versus the HL ratios of Per1 expression in AVP cells in the SCN at 9 h, 6 h, 3 h or 0 h before AO. Similar plots of asymmetry of the intermediate HCRT field are presented in (B). The p and rho values are indicated within each plot. (TIF) [file pone.0067173.s006.tif]
