# Supplementary material for: Central Control of Circadian Phase in Arousal-Promoting Neurons
Source: PLoS One. 2013 Jun 24;8(6):e67173. doi: 10.1371/journal.pone.0067173 (PMC3691112; doi:10.1371/journal.pone.0067173)
Supplement: Table S1 — Cell counts for Experiment 1. (DOCX) [file pone.0067173.s007.docx]

| **Supplement Table 1. Cell counts for Experiment 1.** | | | | |
| --- | --- | --- | --- | --- |
| **Number of cells counted per SCN** | | | | |
| **Circadian Time** | **# of AVP cells** | **# of Per1 cells** | **# of colabeled cells** | **% of AVP colabeled** |
| **3** | 109 + 14^a^ | 156 + 23 | 78 + 18^b^ | 70 + 11^c^ |
| **9** | 93 + 8 | 145 + 25 | 46 + 23 | 47 + 20 |
| **12.5** | 84 + 25 | 135 + 35 | 20 + 10 | 20 + 5 |
| **22** | 65 + 11 | 134 + 12 | 23 + 10 | 36 + 12 |
| **Number of cells counted per side of the LH/DMH** | | | | |
| **Circadian Time** | **# of HCRT cells** | **# of Per1 cells** | **# of colabeled cells** | **% of HCRT colabeled** |
| **3** | 58 + 9 | 135 + 16 | 45 + 8 | 80 + 6 |
| **9** | 40 + 9 | 154 + 12 | 32 + 6 | 84 + 4 |
| **12.5** | 47 + 7 | 125 + 13 | 40 + 8 | 80 + 6 |
| **22** | 31 + 7 | 82 + 14 | 20 + 6 | 65 + 10 |
| **Number of cells counted per LC** | | | | |
| **Circadian Time** | **# of TH cells** | **# of Per1 cells** | **# of colabeled cells** | **% of TH colabeled** |
| **3** | 42 + 4 | 76 + 7 | 33 + 3 | 80 + 3 |
| **9** | 35 + 3 | 78 + 5 | 30 + 2 | 88 + 2 |
| **12.5** | 35 + 3 | 72 + 7 | 28 + 3 | 78 + 3 |
| **22** | 34 + 3 | 69 + 6 | 29 + 3 | 85 + 3 |

Mean + SEM are presented for region specific cell counts. ^a^ P<0.05 vs. CT22, ^b^ p<0.05 vs. CT12.5 and CT22, ^c^ p<0.05 vs. CT12.5.
