# Supplementary material for: Central Control of Circadian Phase in Arousal-Promoting Neurons
Source: PLoS One. 2013 Jun 24;8(6):e67173. doi: 10.1371/journal.pone.0067173 (PMC3691112; doi:10.1371/journal.pone.0067173)
Supplement: Table S2 — Cell counts for Experiment 2. (DOCX) [file pone.0067173.s008.docx]

| **Supplement Table 2. Cell counts for Experiment 2.** | | | | | | | | | | | | | | |
| --- | --- | --- | --- | --- | --- | --- | --- | --- | --- | --- | --- | --- | --- | --- |
| **Number of cells counted per side of the rostral SCN** | | | | | | | | | | | | | | |
| **Hours before AO** |  | **# of AVP cells** | | | | **# of Per1 cells** | | | | | **# of colabeled cells** | | | |
|  | ***Side*** | ***High^a^*** | | | ***Low^b^*** | | ***High^a^*** | | | ***Low^b^*** | | ***High^a^*** | | ***Low^b^*** |
| **9** |  | 39 + 5 | | 39 +7 | | 65 + 6 | | | 74 +7 | | 25 + 4 | | | 25 + 5 |
| **6** |  | 27 + 3 | | 26 + 3 | | 56 + 5 | | | 53 + 4 | | 21 + 2 | | | 20 + 3 |
| **3** |  | 39 + 6 | | 36 +5 | | 68 + 10 | | | 67 + 8 | | 26 + 5 | | | 22 + 3 |
| **0** |  | 45 + 5 | | 42 + 4 | | 70 + 3 | | | 80 + 11 | | 26 + 4 | | | 21 + 3 |
| **Control** |  | 39 + 10 | | 45 + 14 | | 61 + 8 | | | 65 + 9 | | 28 + 6 | | | 33 + 9 |
| **Number of cells counted per side of the middle SCN** | | | | | | | | | | | | | | |
| **9** |  | 38 + 6 | | 41 + 6 | | 75 + 9 | | | 68 + 7 | | 27 + 4 | | | 28 + 4 |
| **6** |  | 28 + 2 | | 29 + 2 | | 59 + 5 | | | 64 + 6 | | 21 + 2 | | | 23 + 1 |
| **3** |  | 36 + 5 | | 35 + 6 | | 66 + 8 | | | 81 + 15 | | 23 + 4 | | | 21 + 5 |
| **0** |  | 39+ 5 | | 43 + 5 | | 67 + 8 | | | 68 + 4 | | 28 + 2 | | | 28 + 2 |
| **Control** |  | 38 + 6 | | 35 + 9 | | 61 + 8 | | | 65 + 9 | | 22 + 5 | | | 21 + 8 |
| **Number of cells counted per side of the caudal SCN** | | | | | | | | | | | | | | |
| **9** |  | 44 + 11 | | 48 + 12 | | 73+ 9 | | | 67 + 11 | | 28 + 4 | | | 34 + 9 |
| **6** |  | 29 + 5 | | 29 + 7 | | 59 + 5 | | | 54 + 6 | | 22 + 3 | | | 19 + 3 |
| **3** |  | 35 + 9 | | 32 + 7 | | 61 + 12 | | | 66 + 11 | | 18 + 3 | | | 17 + 4 |
| **0** |  | 36 + 4 | | 37 + 6 | | 68 + 5 | | | 65 + 7 | | 17 + 3 | | | 19 + 2 |
| **Control** |  | 37 + 2 | | 45 + 10 | | 52 + 10 | | | 81 + 11 | | 20 + 1 | | | 28 + 18 |
| **Number of cells counted per tissue at the level of the LH/DMH** | | | | | | | | | | | | | | |
| **Hours before AO** |  | **# of HCRT cells** | | | | **# of Per1 cells** | | | | | **# of colabeled cells** | | | |
|  | ***Side*** | ***Ipsilateral^c^*** | ***Contralateral^d^*** | | | | | ***Ipsilateral^c^*** | ***Contralateral^d^*** | | | | ***Ipsilateral^c^*** | ***Contralateral^d^*** |
| **9** |  | 35 + 7 | 27 + 5 | | | | | 245 + 36 | 232 + 34 | | | | 35 + 7 | 27 + 5 |
| **6** |  | 24 + 3 | 27 + 4 | | | | | 218 + 23 | 245 + 35 | | | | 24 + 3 | 27 + 4 |
| **3** |  | 25 + 3 | 26 + 8 | | | | | 219 + 35 | 242 + 31 | | | | 25 + 3 | 26 + 8 |
| **0** |  | 24 + 8 | 27 + 7 | | | | | 141 + 35 | 137 + 46 | | | | 24 + 8 | 27 + 7 |
| **Control** |  | 26 + 3 | 25 + 4 | | | | | 238 + 34 | 229 + 20 | | | | 26 + 3 | 25 + 4 |
| **Number of cells counted per tissue at the level of the LC** | | | | | | | | | | | | | | |
| **Hours before AO** |  | **# of TH cells** | | | | | | **# of Per1 cells** | | | | | **# of colabeled cells** | |
|  | ***Side*** | ***Ipsilateral^c^*** | ***Contralateral^d^*** | | | | | ***Ipsilateral^c^*** | ***Contralateral^d^*** | | | | ***Ipsilateral^c^*** | ***Contralateral^d^*** |
| **9** |  | 31 + 4 | 27 + 3 | | | | | 90 + 12 | 78 + 11 | | | | 20 + 3 | 18 + 2 |
| **6** |  | 25 + 2 | 25 + 1 | | | | | 103 + 22 | 98 + 11 | | | | 20 + 2 | 21 + 2 |
| **3** |  | 21 + 2 | 22 + 4 | | | | | 93 + 10 | 92 + 10 | | | | 14 + 2 | 16 + 3 |
| **0** |  | 32 + 4 | 27 + 2 | | | | | 101 + 16 | 89 + 13 | | | | 21 + 3 | 19 + 2 |
| **Control** |  | 32 + 5 | 29 + 3 | | | | | 100 + 9 | 91 + 11 | | | | 25 + 4 | 19 + 2 |

^a^ Number of cells counted on the side of the brain with the higher mean *Per1* signal intensity. ^b^ Number of cells counted on the side of the brain with lower mean *Per1* intensity. ^c^ Number of cells on the side of the brain ipsilateral to the high *Per1* expressing SCN. ^d^ Number of cells on the side of the brain contralateral to the high *Per1* expressing SCN.
